# Fontan associated protein-losing enteropathy is linked to distinct metabolic and hepatic alterations

**DOI:** 10.1038/s41598-026-37974-1

**Published:** 2026-02-05

**Authors:** Christian Schroeder, Fabian B. Fahlbusch, Robert Cesnjevar, Manfred Rauh, Sven Dittrich, Julia Moosmann

**Affiliations:** 1 Department of Pediatric Cardiology, Friedrich-Alexander Universität Erlangen-Nürnberg (FAU), Erlangen, Germany; 2 Division of Neonatology and Pediatric Intensive Care Medicine, Medical Faculty, University of Augsburg, Augsburg, Germany; 3 Department of Cardiac Surgery, Friedrich-Alexander Universität Erlangen-Nürnberg (FAU), Erlangen, Germany; 4 Department of Pediatrics and Adolescent Medicine, Friedrich-Alexander-University of Erlangen-Nürnberg (FAU), Erlangen, Germany; 5 Department of Congenital Heart Disease-Pediatric Cardiology, Deutsches Herzzentrum der Charité (DHZC), Berlin, Germany; 6 Charité - Universitätsmedizin Berlin, Corporate Member of Freie Universität Berlin and Humboldt-Universität zu Berlin, Charitéplatz 1, 10117 Berlin, Germany; 7 German Heart Center of the Charite, Augustenburger Platz 1, 13353 Berlin, Germany

**Keywords:** Fontan circulation, Protein-losing enteropathy (PLE), Targeted metabolomics, Dyslipidemia, Phosphatidylcholine, Cholesterol, Bile acids, Hepatic dysfunction, Renin-angiotensin-aldosterone-system activation, Glycerophospholipids., Biochemistry, Biomarkers, Diseases, Gastroenterology, Medical research

## Abstract

The univentricular Fontan circulation is associated with long-term multiorgan complications, including protein-losing enteropathy (PLE). While hemodynamic and lymphatic contributors to PLE have been described, its systemic metabolic signature remains incompletely characterized. We aimed to identify PLE-associated alterations in circulating metabolites using targeted serum metabolomics. Targeted serum metabolomic profiling was performed by liquid chromatography–tandem mass spectrometry (LC–MS/MS) using the AbsoluteIDQ p180 kit. Forty-nine individuals were included: Fontan patients with PLE (FPLE, *n* = 10), Fontan patients without PLE (F, *n* = 30), and clinically stable biventricular controls (C, *n* = 9). Data were analyzed using MetaboAnalyst v6.0, including multivariate modeling (PLS-DA), univariate statistics with false discovery rate correction, correlation analyses, and receiver operating characteristic (ROC) analyses. Compared with controls, Fontan patients without PLE showed reduced concentrations of cholesterol, triacylglycerols, and several phosphatidylcholine (PC) species, whereas Fontan patients with PLE demonstrated relative increases in these lipid classes. Among 90 quantified PCs, 11 showed a consistent gradient with the lowest concentrations in F and the highest in FPLE. FPLE was further characterized by marked hypoalbuminemia and hypogammaglobulinemia, accompanied by elevated renin, aldosterone, and copeptin levels, indicating pronounced renal–neurohormonal activation of the renin-angiotensin-aldosterone system (RAAS) and vasopressin. Bile acid derivatives, including taurodeoxycholic acid and glycodeoxycholic acid, tended to be lower in FPLE and showed group-specific associations with both renin and selected PC species. Exploratory ROC-based screening identified the immunoglobulin G (IgG)-to-aldosterone and the albumin-to-PC ae C40:3 ratios as the most informative biomarker combinations distinguishing FPLE from non-PLE Fontan patients. These findings are exploratory and hypothesis-generating and require validation in independent cohorts. Fontan patients with PLE show a distinct metabolic phenotype integrating protein loss, lipid alterations, bile acid perturbations, and activation of the renin–angiotensin–aldosterone system. These findings suggest that metabolic and renal–neurohormonal pathways extend beyond lymphatic dysfunction in PLE and identify candidate biomarker patterns for further investigation rather than established diagnostic tools. Further studies are required to clarify causality, mechanistic links, and clinical generalizability.

**Supplementary Information:**

The online version contains supplementary material available at 10.1038/s41598-026-37974-1.

## Introduction

The Fontan procedure is the standard surgical approach for palliation of congenital single-ventricle heart defects^1^. Since its first description in 1971^2^, it has significantly improved survival and outcomes for patients with complex congenital heart disease (CHD)^3,4^. However, the absence of a sub-pulmonary ventricle leads to chronically elevated central venous pressure, non-pulsatile pulmonary blood flow, and reduced cardiac output^5–9^. As a result, the Fontan circulation predisposes patients to a variety of complications, many of which extend beyond circulatory failure^10^. Multiorgan complications are common, with significant impact on morbidity arising from conditions such as protein-losing enteropathy (PLE), plastic bronchitis, lymphatic malformations, renal dysfunction and Fontan-associated liver disease (FALD). The various complications frequently overlap, making clinical management more complex. FALD, particularly when accompanied by liver fibrosis and cirrhosis, is known to increase hepatic lymph production and promote lymphangiogenesis^15,16^. This pathophysiological process is believed to cause characteristic abdominal lymphatic malformations which are seen in patients with PLE^17,18^.

Omic technologies—including genomics, transcriptomics, proteomics, and metabolomics—have significantly transformed our understanding of cardiovascular diseases, such as heart failure, by facilitating the discovery of novel biomarkers and therapeutic targets^11^. Previous studies characterizing metabolic changes in Fontan patients have primarily focused on amino acid and phospholipid metabolomics in adult patients with a dominant left ventricle^12,13^. These prior reports have identified disruptions in cellular signalling and energy metabolism, along with evidence of oxidative stress, chronic low-level inflammation, endothelial dysfunction, as well as structural and/or functional changes in lymphatic vessels^12–14^. Expanding on these findings, Fontan-specific alterations in tricarboxylic acid (TCA) cycle metabolites, including 2-oxoglutaric acid and cis-aconitic acid^14^ could be identified.

However, metabolomic signatures specifically linked to PLE - a complication marked by multisystem involvement and end-organ dysfunction - remain poorly characterized. Therefore, we performed a targeted metabolomic analysis to characterize metabolic differences among three groups: Fontan patients with PLE (FPLE), Fontan patients without PLE (F), and biventricular controls (C). Identifying distinct metabolic patterns in pediatric patients with FPLE, a subgroup at high risk for severe complications and adverse outcomes, may uncover novel pathophysiological mechanisms and reveal novel biomarkers as potential therapeutic targets.

## Materials and methods

### Patients

Patients who had undergone Fontan surgery at the Department of Pediatric Cardiac Surgery and were receiving postoperative follow-up care at the Department of Pediatric Cardiology, University Hospital of Erlangen, Germany, were prospectively invited to participate in this study. Thirty Fontan patients without PLE and ten Fontan patients with PLE consented to participate. As a biventricular control group, nine age-matched patients with dextro-transposition of the great arteries (d-TGA) after arterial switch operation were included, as both control and Fontan groups required neonatal cardiac surgery. d-TGA controls were in good clinical condition, had normal ventricular function, no clinically relevant valvular disease, and without signs of heart failure (NYHA functional class I) at the time of sampling. Patients with residual hemodynamically significant lesions, advanced heart failure, acute intercurrent illness, or conditions known to substantially affect lipid or bile acid metabolism were excluded. Patient characteristics of all three groups are summarized in Table 1. PLE was diagnosed in accordance with the American Heart Association (AHA) Clinical Statement, defining PLE as a clinical syndrome characterized by elevated fecal alpha-1-antitrypsin (> 400 µg/g), hypoalbuminemia (< 30 g/L) or reduced serum total protein (< 50 g/L), and evidence of third-space fluid retention (e.g., ascites, edema, or pleural effusions) in the absence of another identifiable cause^7^.

**Table 1:** Patient demographics, vital signs, and routine laboratory parameters.

	Fontan with PLE (FPLE; *n*=10)	Fontan without PLE (F; *n*=30)	d-TGA (C; *n*=9)	*p*-Value			FDR
				FPLE - F	FPLE - C	F - C	
Demographics							
Female sex	5 (50%)	12 (40%)	2 (22%)	-	-	-	-
Age at examination (years)	13.8 ± 7.86	14.03 ± 6.9	16.22 ± 5.26	ns	ns	ns	ns
Height (cm)	134.23 ± 25.12	155 ± 30.22	150.44 ± 38.92	ns	ns	ns	ns
Weight (kg)	33.07 ± 14.50	49.75 ± 25.10	70.94 ± 44.47	ns	ns	ns	ns
Body surface area (m^2^)	1.1 ± 0.35	1.44 ± 0.51	1.61 ± 0.31	ns	ns	ns	ns
Anatomical and Surgical Details							
Dominance of right ventricle	5 (50%)	12 (40%)	-	ns	-	-	-
Age at TCPC (years)	4.58 ± 5.48	3.68 ± 2.23	-	ns	-	-	-
Time since TCPC (years)	6.89 ± 4.84	9.68 ± 6.43	-	ns	-	-	-
Fenestration	2 (20%)	3 (10%)	-	ns	-	-	-
Vital Signs							
SpO_2_ (%)	93.3 ± 2.36	94.1 ± 4.67	98 ± 2.06	ns	0.013	ns	ns
Heart rate (bpm)	95.3 ± 16.31	80.14 ± 16.14	69.78 ± 13.3	ns	< 0.001	ns	ns
Blood pressure (mmHg)							
Systolic	104.13 ± 8.27	115.83 ± 12.43	124.89 ± 17.66	ns	< 0.0001	ns	0.041
Diastolic	62 ± 5.51	63.41 ± 7.77	60.31 ± 8.5	ns	ns	ns	ns
Mean	76.59 ± 6.02	82.62 ± 8.12	82.57 ± 11.78	ns	ns	ns	ns
Routine Blood Analysis							
Hemoglobin (g/dl)	14.56 ± 2.24	14.57 ± 1.67	14.16 ± 1.86	ns	ns	ns	ns
Erythrocyte count (pl^-1^)	5.1 ± 0.77	4.99 ± 0.54	4.95 ± 0.43	ns	ns	ns	ns
Platelet count (nl^-1^)	259.4 ± 115.33	181.28 ± 62.56	218.78 ± 43.76	0.006	ns	ns	ns
Leucocyte count (nl^-1^)	7.06 ± 2.45	5.32 ± 1.65	6.04 ± 1.07	0.01	ns	ns	ns
Lymphocyte count (nl^-1^)	1.03 ± 0.89	1.34 ± 0.53	1.81 ± 0.35	ns	0.007	0.045	ns
Neutrophile count (nl^-1^)	5.56 ± 1.62	3.11 ± 0.91	3.53 ± 0.94	< 0.001	< 0.001	ns	0.007
Eosinophile count (nl^-1^)	0.26 ± 0.25	0.14 ± 0.07	0.19 ± 0.15	0.026	ns	ns	ns
Basophile count (nl^-1^)	0.05 ± 0.06	0.04 ± 0.04	0.03 ± 0.02	ns	ns	ns	ns
Monocyte count (nl^-1^)	0.66 ± 0.32	0.52 ± 0.22	0.5 ± 0.11	ns	ns	ns	ns
Osmolarity (mOsm/kg)	279.1 ± 3.87	282.07 ± 3.58	284.44 ± 2.07	0.022	0.001	ns	0.043
Sodium (mM)	134.4 ± 1.96	136.2 ± 1.75	137.56 ± 1.01	0.005	< 0.001	0.04	0.013
Potassium (mM)	3.36 ± 0.52	3.99 ± 0.66	3.71 ± 0.24	0.005	ns	ns	0.041
Calcium (mM)	2.1 ± 0.18	2.29 ± 0.17	2.43 ± 0.34	0.015	0.001	ns	0.041
Creatinine (mg/dl)	0.48 ± 0.19	0.62 ± 0.24	0.73 ± 0.23	ns	0.023	ns	ns
Glucose (mg/dl)	93.4 ± 21.84	90.27 ± 16.55	93.22 ± 10.94	ns	ns	ns	ns
Routine Urine Analysis							
Osmolarity (mOsm/kg)	403 ± 201.51	673.33 ± 290.36	811 ± 281.56	0.01	0.002	ns	ns
Sodium (mM)	43.19 ± 40.2	18.58 ± 14.13	12.81 ± 5.64	0.003	0.003	ns	ns
Fractional excretion (%)	1.32 ± 1.08	0.65 ± 0.36	0.59 ± 0.26	0.003	0.008	ns	ns
Potassium (mM)	21.77 ± 20.28	9.97 ± 8.71	6.87 ± 2.23	0.007	0.006	ns	ns
Fractional excretion (%)	87.59 ± 208.29	11.94 ± 9.83	11.63 ± 4.09	0.032	ns	ns	ns
Creatinine (mg/dl)	34.94 ± 30.19	103.78 ± 73.18	148.94 ± 63.68	0.006	< 0.001	ns	0.004
Glucose (mg/dl)	3.6 ± 3.37	7.2 ± 5.02	7.89 ± 6.15	ns	ns	ns	ns

Values are presented as mean ± standard deviation (SD). Metric data were analyzed using one-way ANOVA with Fisher’s least significant difference (LSD) post hoc test; pairwise comparisons used Student’s t-test. Categorical data were analyzed using the Chi-square test. Abbreviations: TCPC = total cavo-pulmonary connection, ns = not significant.

### Sample collection and preparation

Serum samples for metabolomic and biomarker analyses were obtained during peripheral venous blood sampling performed for clinical care, including complete blood count, coagulation parameters, electrolytes, and liver and renal function tests. Clean-catch urine samples were collected for the analysis of electrolytes, osmolality, creatinine, and protein. All routine laboratory analyses were performed at the certified clinical laboratory of the University Hospital of Erlangen, Germany.

### Metabolomic and enzyme-linked immunosorbent assay (ELISA) analyses

Serum samples were centrifuged and stored at -80 °C within 20 min after collection. Targeted metabolomic profiling was performed using the AbsoluteIDQ p180 kit (Biocrates Life Sciences, Innsbruck, Austria) according to the manufacturer’s instructions, as previously described^15^. The kit quantifies 188 metabolites across multiple analyte classes, including acylcarnitines, amino acids, biogenic amines, phosphatidylcholines, sphingolipids, and hexose. Serum bile acids were quantified using targeted metabolomics. Bile acids were categorized into primary and secondary species according to established definitions. Individual bile acid species were analyzed in relation to clinical and biochemical parameters. Enzyme-linked immunosorbent assays (ELISAs) were used to quantify vascular and inflammatory markers, including vascular endothelial growth factor A, C, and D (VEGF-A, VEGF-C, VEGF-D), soluble intercellular adhesion molecule-1 (sICAM-1), neopterin, and monocyte chemoattractant protein-1 (MCP-1). These markers were selected based on their established roles in lymphangiogenesis, endothelial activation, and immune activation relevant to Fontan physiology and PLE^16–20^. All ELISAs were performed according to the manufacturer’s instructions (R&D Systems, Minneapolis, USA).

### Statistical analysis

Statistical analyses were conducted using MetaboAnalyst 6.0, R statistical software version 4.3.0^21,22^, and IBM SPSS Statistics version 29.0.1.0^23^. Prior to analysis, metabolomic data were log10-transformed and Pareto-scaled^24^. Values below the limit of detection were imputed as LOD/2, values below the lower limit of quantification were set to (LOD + LLOQ)/2, and values exceeding the upper limit of quantification were capped at the ULOQ.

#### Univariate analyses and multiple-testing correction

Overall group differences among Fontan patients with PLE, Fontan patients without PLE, and controls were assessed using analysis of variance (ANOVA). Resulting p-values were adjusted for multiple testing using the Benjamini–Hochberg false discovery rate (FDR) procedure, with statistical significance defined as an FDR-adjusted p-value < 0.05. Post-hoc comparisons were used to summarize group-wise means and standard deviations. Categorical variables were analyzed using the chi-squared test, and correlations between continuous variables were assessed using Pearson’s correlation coefficient.

#### Exploratory multivariate analysis

To explore multivariate metabolic patterns and group separation, supervised partial least squares–discriminant analysis (PLS-DA) was performed using MetaboAnalyst version 6. Prior to multivariate modelling, the input feature set was restricted to a limited subset of metabolites selected based on biological plausibility and evidence from univariate analyses, in order to reduce dimensionality and limit model complexity. The maximum number of latent components was limited to five.

Model performance was assessed using five-fold cross-validation, with Q² used as the primary performance metric. Permutation testing was applied to evaluate the robustness of group separation. Variable importance in projection (VIP) scores were calculated to summarize the relative contribution of individual metabolites to group separation across model components, accounting for explained variance^25,26^. Given the limited sample size and exploratory design of the study, PLS-DA was used exclusively as a descriptive pattern-recognition approach and was not interpreted as confirmatory evidence of discrimination.

#### Exploratory ROC-based screening of candidate ratios

Univariate ROC curve analyses were performed as an exploratory screening step to identify candidate metabolic and routine laboratory ratios that discriminate Fontan patients with PLE from those without PLE. ROC curves were derived from the same dataset used to nominate and characterize these ratios. For each analysis, sensitivity, specificity, area under the curve (AUC), and 95% confidence intervals were calculated^27^. Because marker nomination and performance estimation were conducted within the same dataset and the number of patients with PLE was limited (*n* = 10), no internal validation procedures (e.g., bootstrapping or cross-validation) were applied. Accordingly, reported AUC values represent potentially optimistic estimates and should be interpreted as hypothesis-generating rather than confirmatory.

#### Multivariate linear analysis of biomarker ratios

To evaluate whether group differences in the IgG-to-aldosterone and albumin-to-PC ae C40:3 ratios persisted after adjustment for clinical covariates, multivariate linear models were applied. Given strong correlations among age at visit, body size, and time since total cavo-pulmonary connection (TCPC), model parsimony was prioritized to minimize multicollinearity (Supplementary Table 1, Supplementary Fig. 1). A comprehensive model including age at follow-up, years since TCPC, sex, body surface area (BSA), and diuretic therapy, as well as PLE status was compared with reduced models (Supplementary Figure S2). Likelihood ratio testing supported a simplified specification retaining sex, BSA, and diuretic therapy as fixed effects.

Separate models were fitted for each biomarker ratio. Estimated marginal means were calculated to visualize adjusted group differences under diuretic use and non-use.

### Data availability

The original contributions presented in this study are included in the article and its supplementary material. Further information is available from the corresponding author upon reasonable request.

### Ethics

The collection and analysis of human blood samples and clinical data were approved by the Ethics Committee of the University of Erlangen (Re.-No. 145_13B). Written informed consent was obtained from all participants or their legal guardians. The study was conducted in accordance with the Declaration of Helsinki (2024)^28^.

## Results

### Baseline characteristics and descriptive clinical comparisons

A total of 49 patients were included in the analysis, comprising 30 Fontan patients without PLE (F), 10 Fontan patients with PLE (FPLE), and 9 clinically stable biventricular controls (C). Baseline demographic characteristics, anatomical and surgical details, medication use, vital signs, and routine blood and urine laboratory parameters are summarized in Table 1. Overall, demographic variables, cardiac anatomy, and surgical characteristics were comparable between Fontan patients with and without PLE, providing a stable clinical framework for subsequent metabolomic and biomarker analyses. Diuretic therapy was more frequently used in patients with PLE (8/10) compared with Fontan patients without PLE (2/30), whereas beta-blocker use was rare and anticoagulation was common in both Fontan groups (FPLE: 7/10 and Fontan 15/30); none of the biventricular control patients received these medications.

### Univariate group comparisons of metabolites and routine laboratory parameters

#### Protein metabolism, immunoglobulins, and amino acid profiles

Univariate analyses revealed pronounced alterations in protein metabolism and immunoglobulin homeostasis in FPLE (Table 2, Protein Metabolism). After correction for multiple testing, fecal alpha-1-antitrypsin remained significantly elevated in FPLE, while serum concentrations of total protein, albumin, immunoglobulin G (IgG), and immunoglobulin A (IgA) were significantly reduced compared with both Fontan patients without PLE and biventricular controls (FDR < 0.05 for all). Among amino acids and biogenic amines, histidine concentrations were significantly lower, whereas ornithine concentrations were significantly higher in FPLE after FDR correction (Table 2, Amino Acids and Biogenic Amines). In contrast, spermidine showed numerically higher concentrations in FPLE but did not remain significant after adjustment for multiple testing.

**Table 2:** Altered metabolomic pathways and systemic parameters.

	Fontan with PLE (FPLE; *n*=10)	Fontan without PLE (F; *n*=30)	d-TGA (C; *n*=9)	*p*-Value			FDR
				FPLE - F	FPLE - C	F - C	
Protein Metabolism							
Total protein serum (g/l)	48.3 ± 12.21	66.7 ± 4.36	67.2 ± 4.24	< 0.001	< 0.001	ns	ns
Total protein urine (g/l)	162.3 ± 177.29	171.33 ± 124.79	95.33 ± 68.26	ns	ns	ns	ns
Albumin serum (g/dl)	2.88 ± 0.82	4.3 ± 0.27	4.42 ± 0.19	< 0.001	< 0.001	ns	ns
Albumin urine (mg/dl)	4.65 ± 0.83	16.77 ± 22.73	25.55 ± 48.31	ns	ns	ns	ns
Faecal alpha-1-antitrypsine (µg/g)	1424.5 ± 640.52	247.93 ± 321.71	238.33 ± 150.48	< 0.001	< 0.001	ns	ns
Faecal Calprotectin (µg/g)	59.06 ± 121.75	24.9 ± 42.58	13.78 ± 22.3	ns	ns	ns	ns
Amino acids							
Histidine (µM)	78.68 ± 16.64	90.53 ± 11.9	108.21 ± 18.3	0.027	< 0.001	0.002	0.007
Ornithine (µM)	141 ± 21.06	101.31 ± 22.29	94.36 ± 21.16	< 0.001	< 0.001	ns	0.004
Arginine (µM)	97.3 ± 16.23	107.66 ± 22.07	124.24 ± 21.28	ns	0.007	ns	ns
Biogenic amines							
Spermidine (µM)	0.28 ± 0.08	0.23 ± 0.05	0.2 ± 0.03	0.01	0.004	ns	ns
Creatinine (µM)	47.74 ± 19.96	64.87 ± 24.72	75.13± 23.46	ns	0.015	ns	ns
Immunoglobulins							
IgG (U/l)	3.66 ± 3.72	9.44 ± 2.7	9.31 ± 2.58	< 0.001	< 0.001	ns	< 0.001
IgA (U/l)	0.67 ± 0.38	1.48 ± 0.8	1.23 ± 0.52	0.003	ns	ns	0.017
Renal Regulation							
Renin (pg/ml)	326.6 ± 675.07	52.21 ± 129.15	23.67 ± 16.07	0.02	0.04	ns	0.017
Angiotensin II (U/l)	51.8 ± 45.62	19.94 ± 26.5	20.02 ± 12.96	0.007	0.026	ns	ns
Aldosterone (ng/ml)	0.29 ± 0.22	0.09 ± 0.09	0.08 ± 0.04	< 0.001	< 0.001	ns	0.004
Copeptin (pmol/l)	16.35 ± 14.33	6.86 ± 5.6	7.84 ± 5.1	0.002	0.026	ns	0.041
Liver Damage							
GGT (U/l)	58.5 ± 45.33	80.73 ± 119.48	18.22 ± 6.53	ns	<0.001	<0.001	0.009
AST (U/l)	6.43 ± 8.62	2.26 ± 1.47	1.28 ± 0.94	ns	0.024	ns	ns
ALT (U/l)	28 ± 10.65	24.33 ± 8.13	16.44 ± 4.61	ns	0.004	0.015	0.041
GLDH (U/I)	6.43 ± 8.62	2.26 ± 1.47	1.28 ± 0.94	ns	0.004	ns	0.041
Lipid and Fatty Acid Metabolism							
Triacylglycerol (mg/dl)	137.37 ± 77.98	65.29 ± 23.79	105.4 ± 59.45	< 0.001	ns	0.028	0.004
Cholesterol (mg/dl)	165.15 ± 33.13	125.48 ± 22.58	145.34 ± 35.43	< 0.001	ns	ns	0.033
Glycerophospholipids (total, mM)	2.44 ± 0.47	1.92 ± 0.26	2.2 ± 0.43	< 0.001	ns	0.041	0.016
lysoPC a C16:0 (µM)	96.65 ± 13.74	86.38 ± 14.24	105.07 ± 18.16	ns	ns	0.002	ns
lysoPC a C18:2 (µM)	21.14 ± 8.68	27.2 ± 8.59	32.94 ± 6.2	0.05	0.003	ns	ns
PC aa C34:1 (µM)	320.3 ± 90.81	238.03 ± 42.76	275.22 ± 48.63	< 0.001	ns	ns	0.016
PC aa C34:2 (µM)	579.7 ± 107.62	445.87 ± 75.28	515 ± 121.01	< 0.001	ns	ns	0.017
PC aa C36:3 (µM)	183.6 ± 60.47	124.63 ± 19.86	147.88 ± 35.63	< 0.001	0.029	ns	0.006
PC aa C36:4 (µM)	212.4 ± 39.43	154.41 ± 31.65	194.26 ± 53.55	< 0.001	ns	0.008	0.009
PC aa C36:5 (µM)	20.94 ± 11.08	13.04 ± 5.14	24.18 ± 14.58	0.018	ns	0.002	0.032
PC aa C38:4 (µM)	108.76 ± 20.58	83.68 ± 19.61	91.10 ± 23.49	0.002	ns	ns	0.043
PC aa C38:5 (µM)	52.52 ± 13.72	36.91 ± 8.62	47.41 ± 15.07	< 0.001	ns	0.016	0.003
PC aa C38:6 (µM)	65.29 ± 20.45	44.33 ± 13.97	62.83 ± 26.17	0.003	ns	0.01	0.043
PC ae C30:1 (µM)	0.04 ± 0.05	0.1 ± 0.05	0.1 ± 0.05	0.004	0.017	ns	ns
PC ae C34:3 (µM)	7.32 ± 2.58	8.89 ± 2.6	10.8 ± 2.14	ns	0.004	ns	0.045
PC ae C36:1 (µM)	9.27 ± 1.92	7.21 ± 1.21	7.87 ± 1.92	< 0.001	0.049	ns	ns
PC ae C36:2 (µM)	17.08 ± 3.78	12.99 ± 2.15	14.33 ± 4.42	< 0.001	ns	ns	ns
PC ae C38:0 (µM)	1.83 ± 0.55	1.29 ± 0.34	1.82 ± 0.78	0.004	ns	0.006	0.041
Sphingolipids (total, mM)	0.16 ± 0.02	0.14 ± 0.02	0.15 ± 0.02	ns	ns	ns	ns
Acylcarnitine (total, mM)	0.05 ± 0.01	0.06 ± 0.01	0.06 ± 0.01	0.032	ns	ns	ns

Comparison of key metabolomic and regulatory features across groups. Metric data were analyzed using one-way ANOVA with Fisher’s LSD post hoc test; pairwise comparisons used Student’s t-test. Abbreviation: ns = not significant.#.

#### Renal-neurohormonal regulation and electrolyte homeostasis

**Fig. 1:**
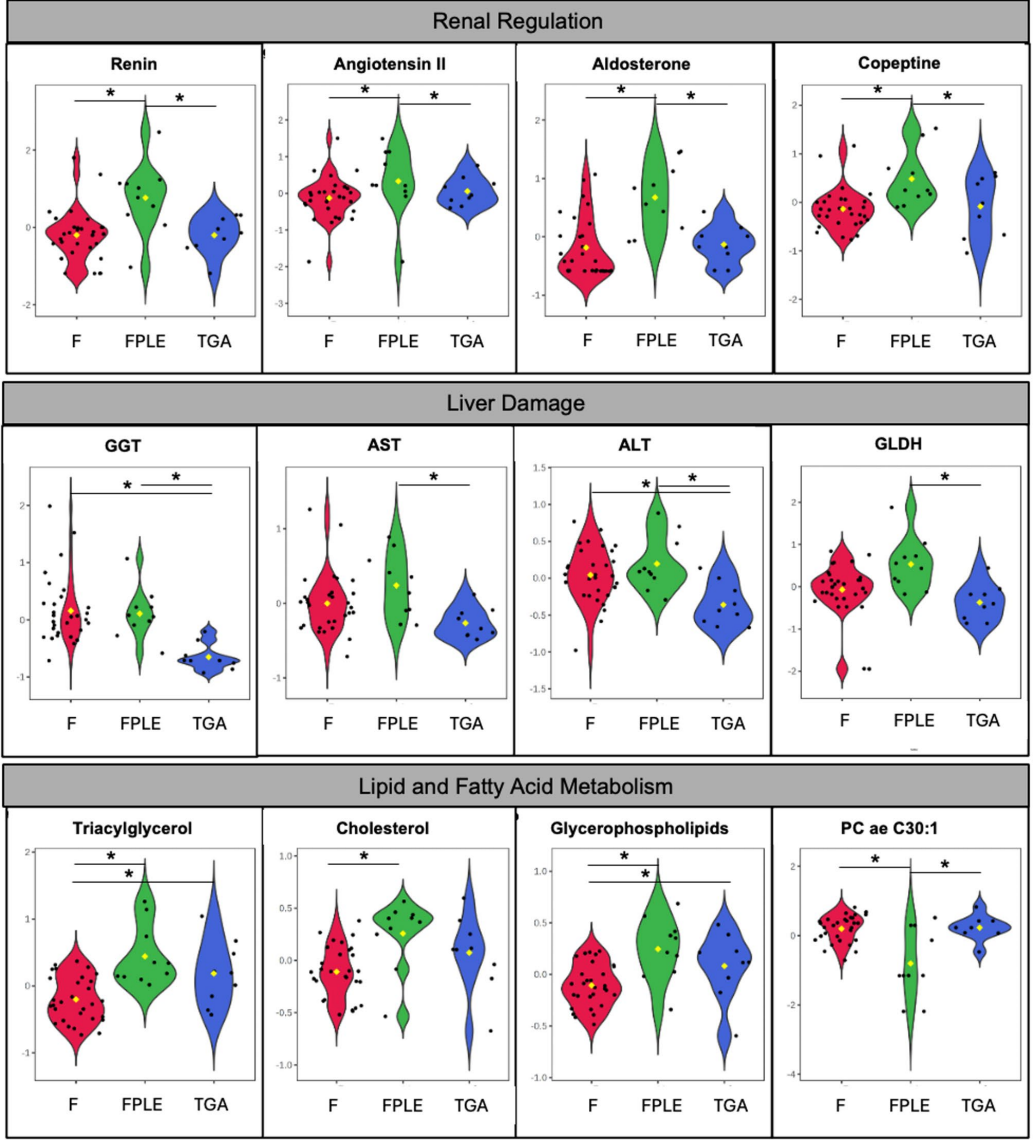
Group-wise comparison of key metabolic features. Normalized metabolite values in Fontan patients without PLE (red), with PLE (green), and biventricular controls with d-TGA (blue), displayed as violin plots. Statistical comparisons were performed on normalized data. Significant differences are indicated by asterisks (*).

Parameters reflecting renal–neurohormonal regulation (renin-angiotensin-aldosterone-system (RAAS)/vasopressin) differed between groups (Tables 1 and 2, Renal Regulation). After FDR correction, markers of renin–angiotensin–aldosterone system activation, including renin, aldosterone, and copeptin, were significantly elevated in FPLE compared with non-PLE Fontan patients and controls (Table 2; Fig. 1, Renal Regulation). Several routine electrolyte parameters showed numerical group differences that did not persist after correction for multiple testing.

#### Hepatic injury markers

Group comparisons of hepatic injury markers demonstrated heterogeneous patterns across cohorts (Table 2, Liver Damage). After FDR correction, gamma-glutamyltransferase (GGT), alanine aminotransferase (ALT), and glutamate dehydrogenase (GLDH) differed significantly between groups, whereas aspartate aminotransferase (AST) did not remain significant (Table 2; Fig. 1, Liver Damage).

#### Lipid and fatty acid metabolism

Univariate lipidomic analyses identified group-dependent alterations across lipid classes (Table 2, Lipid and Fatty Acid Metabolism). After FDR correction, cholesterol, triacylglycerols, and total glycerophospholipids differed significantly between groups (Table 2; Fig. 1, Lipid and Fatty Acid Metabolism). Multiple phosphatidylcholine species (both PC aa and PC ae) exhibited FDR-significant differences, with highest concentrations observed in FPLE and lowest concentrations in Fontan patients without PLE. In contrast, sphingolipids and acylcarnitines did not show FDR-significant group differences.

### Adjusted associations in multivariate linear regression models

#### Group-dependent distributions of biomarker ratios

**Fig. 2:**
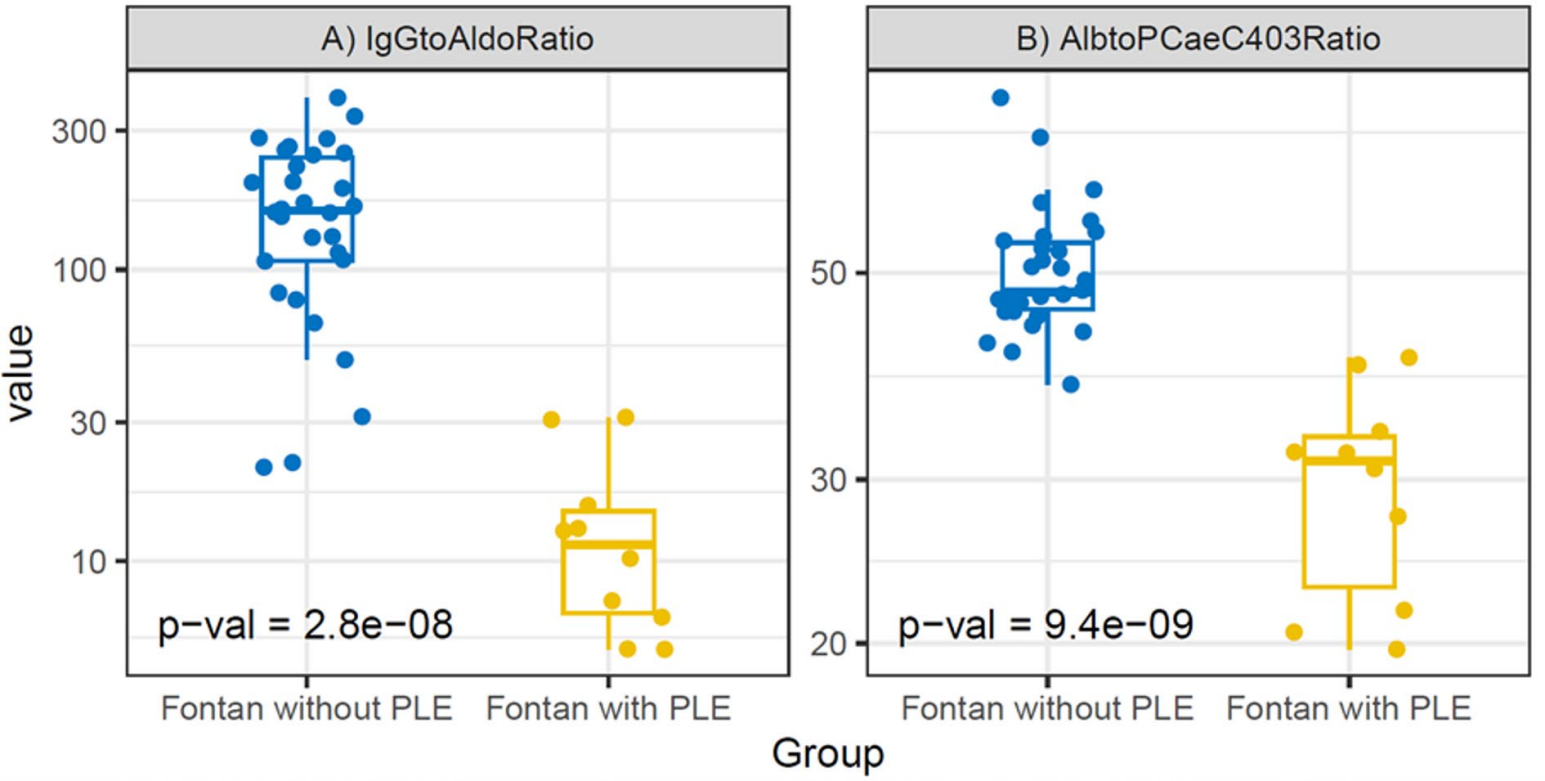
Boxplots illustrating the distribution of (A) IgG-to-aldosterone and (B) albumin-to-PC ae C40:3 ratios in Fontan patients with and without protein-losing enteropathy (PLE). Both biomarker ratios differed between groups in unadjusted analyses.

To translate the univariate alterations in protein metabolism, lipid profiles, and renal–neurohormonal regulation into candidate composite biomarkers, we performed an exploratory ROC-based screening of candidate single markers and ratios. The IgG-to-aldosterone ratio and the albumin-to-PC ae C40:3 ratio showed the highest discriminatory potential (see 3.5) and were therefore examined further. Both ratios showed distinct group-dependent distributions, with lower values observed in Fontan patients with PLE compared with Fontan patients without PLE (Fig. 2).

#### Effect of diuretic therapy and adjusted group differences

Diuretic therapy was used significantly more frequently in FPLE (see 3.1), diuretic use was considered a potential confounder in the analysis of biomarker ratios. Multivariate linear models were therefore applied to assess adjusted group differences while accounting for sex, body surface area, and diuretic therapy. In these models, no relevant association was observed between diuretic use and either the IgG-to-aldosterone ratio or the albumin-to-PC ae C40:3 ratio (Table 3). In contrast, group status (FPLE vs. F patients) accounted for the majority of variability in both ratios. Estimated marginal means confirmed that group differences in both ratios persisted after adjustment for diuretic therapy. Model structure and covariance assumptions are shown in Supplementary Table S1 / Figure S1.

**Table 3:** Results of the simplified multivariate linear models for biomarker ratios.

**Term**	IgGtoAldoRatio		**AlbtoPCaeC403Ratio**	
	Estimate (95% CI)	pval	**Estimate (95% CI)**	pval
SexMale	-22.89 (-79.60-33.83)	0.429	3.38 (-2.35-9.11)	0.248
BSA	47.04 (-12.62-106.70)	0.122	0.28 (-5.74-6.30)	0.928
DiureticsTRUE	-4.14 (-99.07-90.80)	0.932	-4.08 (-13.75-5.59)	0.408

Biomarker ratios were regressed on sex, BSA, diuretic therapy, and PLE status. Legend: Variables included were sex (male vs. female), body surface area (BSA), current diuretic therapy (yes/no), PLE status (with/without), the IgG-to-aldosterone ratio, and the albumin-to-phosphatidylcholine ae C40:3 ratio.

### Exploratory multivariate pattern analysis

**Fig. 3:**
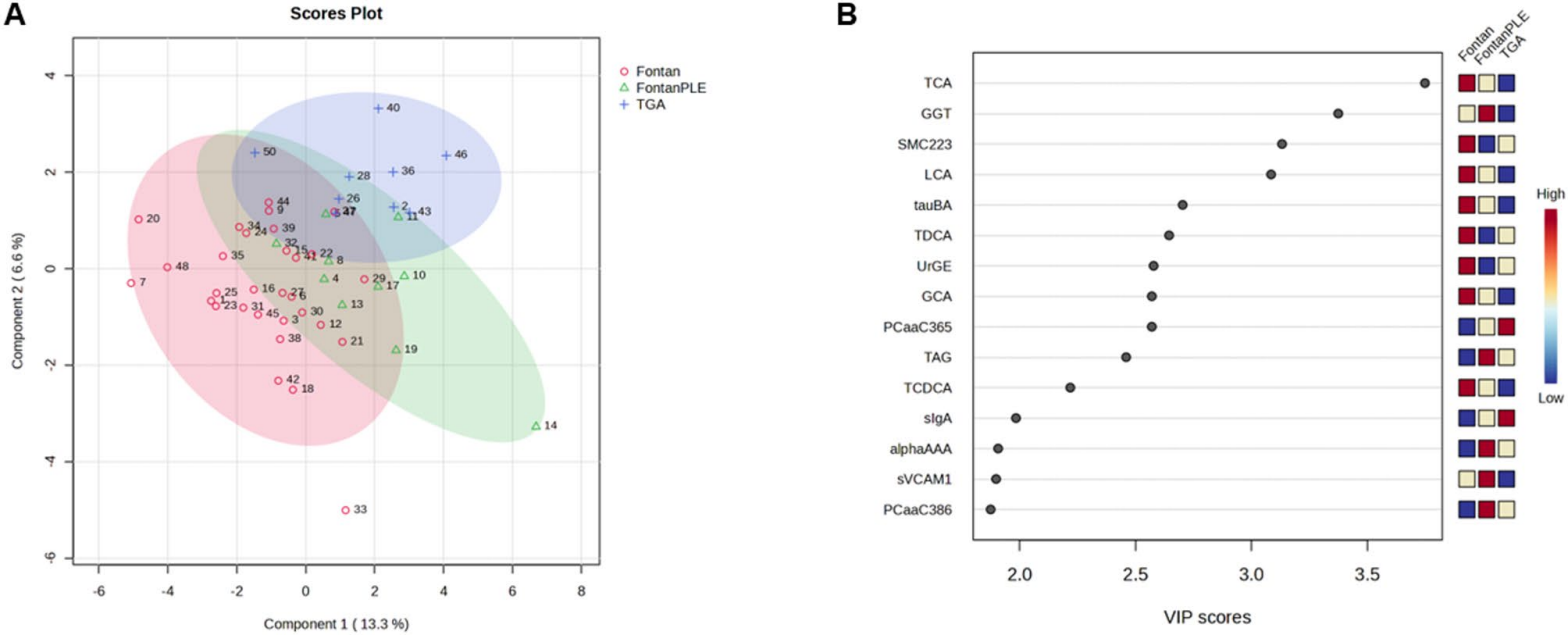
Partial least squares–discriminant analysis (PLS-DA). (**A**) Two-dimensional scores plot illustrating multivariate separation between Fontan patients without protein-losing enteropathy (F), Fontan patients with protein-losing enteropathy (FPLE), and controls (C). Partial overlap between groups reflects inter-individual metabolic variability. (**B**) Variable importance in projection (VIP) scores from the optimized PLS-DA model highlighting variables contributing most strongly to group separation. Variables are color-coded according to relative group means, with higher mean values indicated in red and lower mean values in blue. VIP scores are shown for exploratory pattern recognition rather than inferential testing. Abbreviations: Abbreviations: VIP, variable importance in projection; TCA, taurocholic acid; LCA, lithocholic acid; tauBA, total taurine-conjugated bile acids; TDCA, taurodeoxycholic acid; TCDCA, taurochenodeoxycholic acid; GCA, glycocholic acid; SM C22:3, sphingomyelin (22:3); PC aa C36:5, phosphatidylcholine diacyl (36:5); PC aa C38:6, phosphatidylcholine diacyl (38:6); TAG, triacylglycerol; GGT, gamma-glutamyltransferase; UrGE, urinary gamma-glutamyltransferase equivalent; sIgA, secretory immunoglobulin A; α1A, alpha-1 antitrypsin; sVCAM-1, soluble vascular cell adhesion molecule-1.

PLS-DA was applied as an exploratory multivariate approach. In the scores plot, Fontan patients without PLE and controls occupied largely distinct regions of the multivariate space, whereas FPLE patients clustered in an intermediate position with partial overlap with both groups (Fig. 3A).

**Fig. 4:**
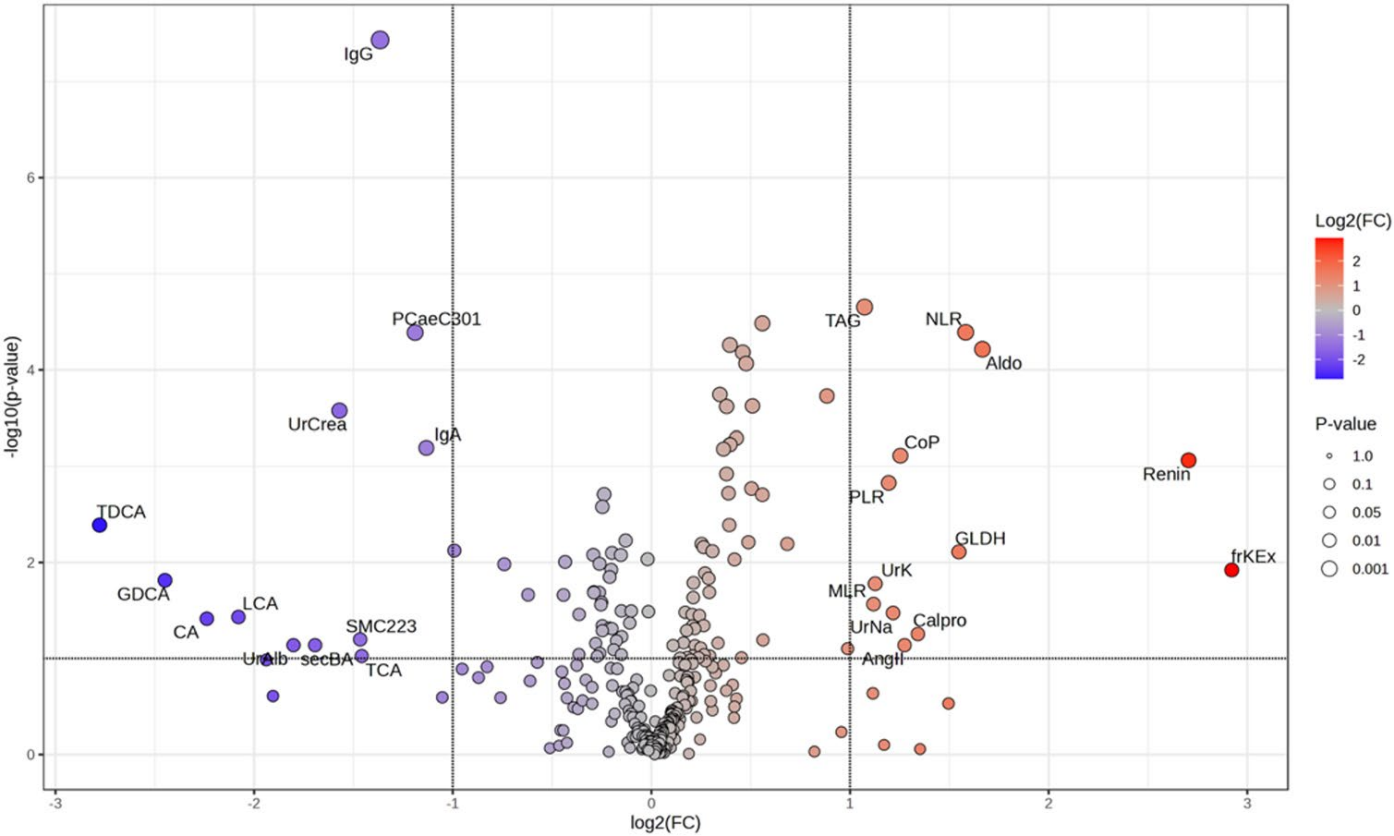
Volcano plot of fold change analysis. Volcano plot depicting metabolites differentially abundant between Fontan patients without PLE and those with PLE. Significant features are highlighted. Abbreviations: Aldo = Aldosterone, Ang II = Angiotensin II, CA = Cholic acid, Calpro = Fecal calprotectin, CoP = Copeptin, frKEx = Fractional potassium excretion (urine), GDCA = Glycodeoxycholic acid, LCA = Lithocholic acid, secBA = Secondary bile acids, MLR = Monocyte-to-lymphocyte ratio, NLR = Neutrophil-to-lymphocyte ratio, PLR = Platelet-to-lymphocyte ratio, TAG = Triacylglycerol, TCA = Taurocholic acid, TDCA = Taurodeoxycholic acid, UrAlb = Urinary albumin, UrCrea = Urinary creatinine, UrK = Urinary potassium, UrNa = Urinary sodium.

Variables contributing most strongly to group separation, as indicated by VIP scores, were predominantly related to lipid and bile acid metabolism, including taurocholic acid, lithocholic acid, total taurine-conjugated bile acids, sphingomyelin SM C22:3, and gamma-glutamyltransferase (Fig. 3B). These multivariate patterns were consistent with the univariate metabolite profiles (Fig. 4; Table 4).

**Table 4:** Fold change analysis comparing Fontan patients with PLE (FPLE) to those without PLE (F).

A) Decrease of absolute values in FPLE compared to F			B) Increase of absolute values in FPLE compared to F		
	Fold Change	*p*-Value		Fold Change	*p*-Value
TDCA	0.146	0.004	frKEx	7.577	0.012
GDCA	0.183	0.015	Renin	6.518	< 0.001
CA	0.212	0.038	Aldo	3.175	< 0.001
LCA	0.237	0.037	NLR	2.994	< 0.001
UrAlb	0.287	0.073	GLDH	2.925	0.008
secBA	0.309	0.073	Calpro	2.535	0.056
UrCrea	0.337	< 0.001	AngII	2.419	0.073
SM C22:3	0.362	0.063	CoP	2.384	< 0.001
TCA	0.364	0.094	UrNa	2.325	0.034
IgG	0.388	< 0.001	PLR	2.289	0.001
PC ae C30:1	0.438	< 0.001	UrK	2.184	0.017
IgA	0.456	< 0.001	TAG	2.104	< 0.001

Fold change and *p*-values are shown for metabolites and clinical parameters with either a decrease (A) or increase (B) in FPLE relative to F. Abbreviations: Aldo = Aldosterone, Ang II = Angiotensin II, CA = Cholic acid, Calpro = Fecal calprotectin, CoP = Copeptin, frKEx = Fractional potassium excretion (urine), GDCA = Glycodeoxycholic acid, LCA = Lithocholic acid, secBA = Secondary bile acids, MLR = Monocyte-to-lymphocyte ratio, NLR = Neutrophil-to-lymphocyte ratio, PLR = Platelet-to-lymphocyte ratio, TAG = Triacylglycerol, TCA = Taurocholic acid, TDCA = Taurodeoxycholic acid, UrAlb = Urinary albumin, UrCrea = Urinary creatinine, UrK = Urinary potassium, UrNa = Urinary sodium, SM = Sphingomyelin, PC ae = Acyl-alkyl phosphatidylcholine.

To contextualize the univariate and adjusted findings, exploratory correlation analyses revealed group-dependent patterns of association between selected bile acids, renin, and phosphatidylcholine species that were predominantly observed in patients with PLE (Supplementary Tables S2-S3).

### Exploratory diagnostic performance of metabolic ratios

**Fig. 5:**
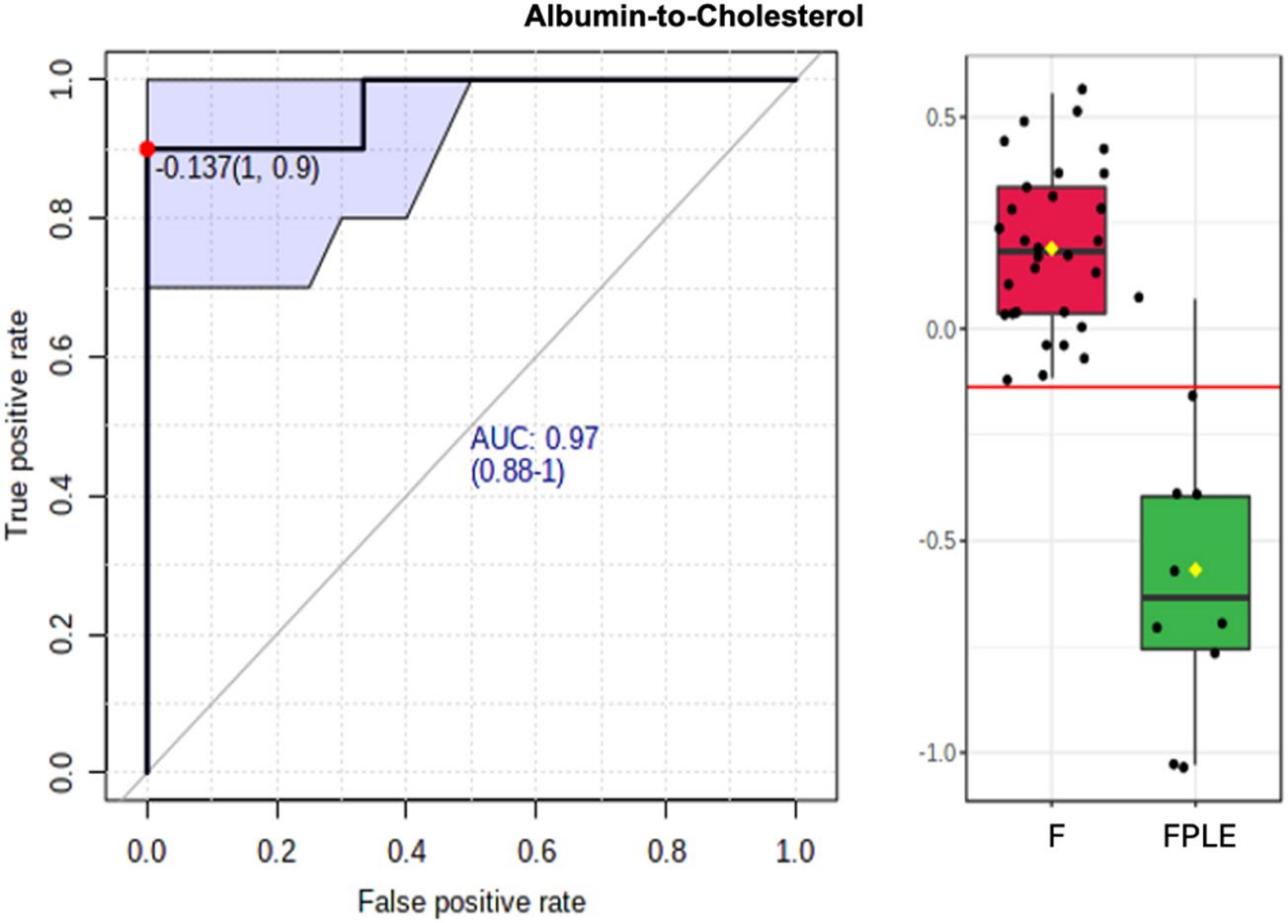
Univariate ROC analysis. Exemplary ROC curve for the albumin-to-PC ae C40:3 ratio illustrating exploratory discriminatory performance between FPLE and non-PLE Fontan patients. Marker nomination and performance estimation were performed within the same dataset without internal validation.

ROC analyses were performed as part of this exploratory screening to compare the discriminatory performance of candidate metabolic and routine laboratory ratios in differentiating Fontan patients with and without PLE. The IgG-to-aldosterone ratio and the albumin-to-PC ae C40:3 ratio showed the highest areas under the curve among the evaluated parameters (Table 5). A representative ROC curve is shown in Fig. 5. Because ratio nomination and performance estimation were performed in the same dataset, AUC estimates should be interpreted as hypothesis-generating and may overestimate true discriminatory performance.

**Table 5:** Univariate ROC analysis of selected biomarker ratios distinguishing Fontan patients with and without PLE.

Neutrophile-to-Lymphocyte	AUC (CI)	Fontan with PLE (*n* = 10)	Fontan wo. PLE (*n* = 30)	*p*-Value
	0.897 (0.7-1)	7.5 ± 4.11	2.58 ± 1.17	< 0.001
Platelets-to-Lymphocyte	0.837 (0.618–0.993)	327.8 ± 209.61	147.72 ± 63.41	0.002
IgG-to-Aldosterone	0.99 (0.96-1)	13.7 ± 9.75	167.55 ± 93.73	< 0.001
IgG-to-Cholesterol	0.917 (0.73-1)	0.02 ± 0.03	0.08 ± 0.03	< 0.001
IgG-to-TAG	0.943 (0.81-1)	0.04 ± 0.04	0.16 ± 0.07	< 0.001
IgG-to-Ornithine	0.927 (0.748-1)	0.03 ± 0.03	0.1 ± 0.03	< 0.001
IgG-to-C18:1	0.96 (0.873-1)	24.04 ± 19.64	81.8 ± 25.09	< 0.001
IgG-to-PC aa C36:3	0.933 (0.79-1)	0.02 ± 0.03	0.08 ± 0.03	< 0.001
IgG-to-PC ae C36:1	0.927 (0.77-1)	0.42 ± 0.48	1.32 ± 0.45	< 0.001
IgG-to-PC ae C44:5	0.953 (0.852-1)	2.89 ± 2.18	8.55 ± 2.75	< 0.001
Albumin-to-Cholesterol	0.97 (0.88-1)	0.18 ± 0.06	0.35 ± 0.07	< 0.001
Albumin-to-PC aa C34:1	0.963 (90.88-1)	0.1 ± 0.04	0.19 ± 0.03	< 0.001
Albumin-to-PC aa C36:3	0.997 (0.967-1)	0.17 ± 0.07	0.35 ± 0.06	< 0.001
Albumin-to-PC aa C36:4	0.963 (0.87-1)	0.14 ± 0.06	0.29 ± 0.06	< 0.001
Albumin-to-PC ae C36:1	0.983 (0.933-1)	3.2 ± 1.02	6.12 ± 1.05	< 0.001
Albumin-to-PC ae C36:2	0.993 (0.958-1)	1.75 ± 0.55	3.41 ± 0.67	< 0.001
Albumin-to-PC ae C38:3	0.977 (0.907-1)	6.55 ± 2.38	12.34 ± 2.17	< 0.001
Albumin-to-PC ae C40:3	0.997 (0.97-1)	28.84 ± 7.46	50.53 ± 8.3	< 0.001
Albumin-to-SM C16:0	0.977 (0.92-1)	0.35 ± 0.08	0.6 ± 0.1	< 0.001

Ratios are presented as mean ± standard deviation (before normalization). ROC analysis was performed on log10-transformed and Pareto-scaled data. Parameters shown represent selected biomarker ratios of interest. ROC p-values refer to testing AUC against 0.5 and are not adjusted for multiple testing. Marker selection and ROC performance estimation were conducted within the same dataset without internal validation. Due to the exploratory nature of the analysis and the limited sample size of the FPLE group, AUC values illustrate discriminatory potential rather than establish diagnostic thresholds and require confirmation in independent cohorts.

### Targeted ELISA analysis highlights vascular and inflammatory pathways

**Fig. 6:**
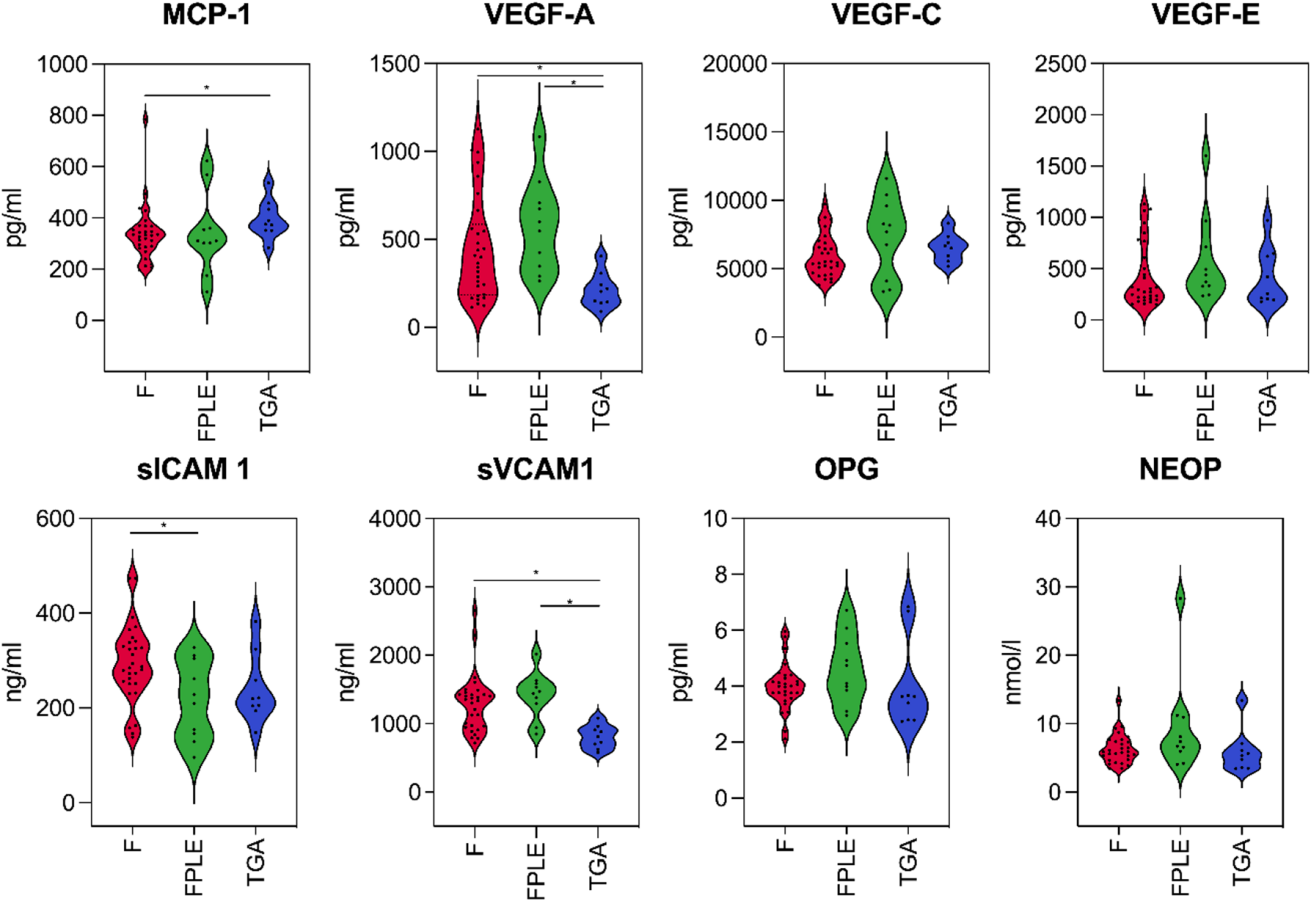
Group-wise comparison of selected biomarkers measured by enzyme-linked immunosorbent assays (ELISAs). Data for Fontan patients without PLE (red), Fontan patients with PLE (green), and biventricular controls with d-TGA (blue) are shown as violin plots. Statistically significant differences are indicated by asterisks (*).

Targeted ELISA analyses revealed distinct patterns of circulating proteins associated with vascular and inflammatory pathways. VEGF-A, a key regulator of angiogenesis, was significantly elevated in both F and FPLE groups (*p* = 0.0039). sVCAM-1, an adhesion molecule facilitating leukocyte migration and a marker of vascular inflammation, was markedly increased in FPLE and F (*p* < 0.0005). A comprehensive overview of all ELISA results is provided in Fig. 6.

## Discussion

### Summary of key results

In this study cohort, PLE was associated with a distinct and internally consistent metabolic and biomarker profile. Univariate analyses identified robust FDR-significant alterations in markers of protein metabolism and immunoglobulin homeostasis, including elevated fecal alpha-1-antitrypsin and reduced serum total protein, albumin, IgG, and IgA. Beyond protein loss, PLE was characterized by activation of renal-neurohormonal regulation, with elevated renin, aldosterone, and copeptin levels compared with Fontan patients without PLE. In parallel, group-specific alterations in lipid metabolism were observed, including differences in multiple glycerophospholipid species, cholesterol, and triacylglycerols, suggesting a broader disturbance of lipid homeostasis associated with FPLE. Adjusted multivariate linear models demonstrated that group status (FPLE vs. F) accounted for the majority of variability in key biomarker ratios, independent of sex, body surface area, and diuretic therapy. Exploratory multivariate analyses further supported these findings by revealing lipid- and bile acid-related metabolic patterns associated with the FPLE phenotype. Together, these results delineate a coherent metabolic phenotype of PLE in the Fontan circulation, integrating disturbances in protein homeostasis, renal-neurohormonal regulation, and lipid-associated metabolic pathways.

### Metabolic characterization of PLE in Fontan circulation

The management of PLE in Fontan patients remains a growing clinical challenge, and early identification of patients at risk is limited by an incomplete understanding of the underlying pathophysiology. In this study, targeted metabolomics combined with routine laboratory parameters was used to characterize metabolic alterations associated with PLE. Previous studies have reported proteomic and metabolomic changes in clinically stable Fontan patients, including alterations in immune system activation and cardiovascular development^16,17,29^. Our findings confirm these systemic metabolic disturbances in Fontan circulation and extend them by identifying a pattern that is specific to patients with PLE, integrating protein loss, activation of renal-neurohormonal regulatory systems (RAAS/vasopressin), and lipid dysregulation. While hepatic impairment was present in both Fontan groups, FPLE patients demonstrated a more pronounced disturbance of protein and lipid metabolism, altered bile acid profiles, and signs of neuroendocrine activation. This constellation suggests that PLE is not an isolated intestinal phenomenon but rather reflects coordinated dysfunction across hepatic, lymphatic, renal, and endocrine systems.

### Adjusted associations of composite biomarker ratios

As diuretic therapy was significantly more frequently used in Fontan patients with PLE and may influence renal-neurohormonal regulation (RAAS/vasopressin), diuretic use was considered a potential confounder in the interpretation of composite biomarker ratios. The higher prevalence of diuretic treatment in this group is clinically plausible as diuretics are a core component of symptomatic therapy in patients with PLE and more advanced Fontan failure. In this exploratory metabolomic analysis, differences in the IgG-to-aldosterone and albumin-to-PC ae C40:3 ratios between Fontan patients with and without PLE were robust across multiple model specifications. Importantly, these group differences persisted after adjustment for diuretic therapy and key clinical covariates, indicating that the observed metabolic signatures are unlikely to be primarily driven by pharmacological treatment effects. A numerically lower IgG-to-aldosterone ratio in patients receiving diuretics is biologically plausible, given the well-established activation of RAAS under diuretic therapy in other clinical settings^30^. However, the absence of a statistically significant association and the persistence of group effects after adjustment argue against a dominant treatment-driven mechanism in the present cohort. The contrast between low marginal R² values for fixed clinical covariates and high conditional R² values in the mixed-effects models suggests that PLE status itself represents the principal source of variability in these composite markers. This supports the interpretation that the investigated ratios capture disease-specific pathophysiological processes rather than general disease severity, body size, or treatment burden. Nonetheless, the limited number of patients with PLE and the wide confidence intervals underscore the exploratory nature of these findings and the need for confirmation in larger, independent cohorts.

### Protein loss, inflammation, and vascular pathways

Consistent with the AHA definition of PLE^7^, FPLE demonstrated pronounced hypoalbuminemia, hypogammaglobulinemia, and elevated fecal alpha-1-antitrypsin, reflecting substantial systemic protein loss. Although fecal calprotectin was not significantly elevated, its high interindividual variability may have masked subtle inflammatory differences^31^.

Beyond protein depletion, our data highlight the involvement of angiogenic and inflammatory vascular pathways. VEGF-A, a mediator of angiogenesis as well as endothelial permeability and inflammation, was elevated in both Fontan groups, consistent with previous reports in Fontan patients^16^. While adhesion molecules that facilitate leukocyte recruitment and intercellular interactions, such as sICAM-1 and sVCAM-1 were increased particularly in patients with FPLE, indicating enhanced systemic endothelial activation. Given established associations of sVCAM-1 with adverse outcomes in heart failure these findings suggest that vascular inflammation and endothelial remodeling may potentially contribute to disease severity and systemic manifestations of PLE^18^.

### Amino acids as markers of systemic metabolic stress

PLE was associated with distinct alterations in circulating amino acid profiles, most notably reduced histidine and elevated ornithine concentrations that remained significant after FDR correction. Ornithine, a central intermediate of the urea cycle and precursor of spermidine, reflects hepatic amino acid handling and nitrogen disposal, and its elevation is consistent with increased amino acid catabolism under conditions of chronic protein loss and systemic stress^32^. While spermidine, known for its pro-inflammatory effects particularly in the gut, showed a numerical increase in FPLE, this difference did not remain significant after FDR correction and should therefore be interpreted cautiously^33^. Reduced histidine concentrations are of particular interest given histidine’s emerging role in immune regulation, oxidative stress modulation, and cardiovascular homeostasis^34^. Beyond its function as a proteinogenic amino acid, histidine availability has been linked to chronic inflammatory states and adverse cardiometabolic profiles, reflecting increased metabolic consumption rather than simple nutritional deficiency^35,36^. As the precursor of histamine, histidine contributes to myocardial stress responses, and experimental as well as clinical data suggest cardioprotective effects of intact histidine-histamine signalling pathways^37,38^. In the context of FPLE, the combination of reduced histidine and elevated ornithine points toward coordinated alterations in amino acid handling, consistent with enhanced nitrogen turnover and hepatic metabolic stress rather than isolated gastrointestinal protein loss^39,40^.

### Hepatic involvement and Fontan-associated liver disease

Markers associated with hepatocellular injury differed between groups, with GGT, ALT, and GLDH being elevated in patients with FPLE and, to a lesser extent, in Fontan patients without PLE compared to controls. While classical transaminases and markers of hepatocellular injury did not consistently indicate overt liver damage, GGT emerged as the most robust liver-associated parameter distinguishing FPLE from non-PLE patients. This observation aligns with data from adult Fontan cohorts, in which standard liver function tests may remain within reference ranges despite ongoing hepatic congestion, metabolic strain, and the risk of developing Fontan-associated liver disease (FALD)^41^.

Although the present cross-sectional design precludes inference on temporal relationships, the observed pattern supports the concept that hepatic metabolic stress accompanies - and may precede - overt manifestations of PLE. Importantly, these alterations should not be interpreted as evidence of advanced hepatobiliary disease but rather as biochemical indicators of systemic and circulatory stress inherent to Fontan physiology^42^.

### Distinct lipidomic signatures and systemic metabolic stress

Consistent with prior reports, Fontan patients without PLE exhibited hypocholesterolemia compared with controls^43^. In contrast, patients with PLE showed higher cholesterol and triacylglycerol levels, suggesting a protein-loss-associated dyslipidemia that has not been systematically characterized in this population. Although apolipoproteins were not measured, the combination of lipid accumulation and protein loss may indicate altered lipid transport or clearance pathways^44,45^. Among glycerophospholipids, multiple phosphatidylcholine species differed significantly between groups and contributed to albumin-based composite ratios. These findings suggest that PLE-associated metabolic disturbances preferentially affect protein and glycerophospholipid handling rather than membrane lipid composition or mitochondrial fatty acid oxidation. In line with this, sphingolipids and acylcarnitines did not differ significantly between groups^46–50^.

### Bile acids, renal–neurohormonal regulation, and neuroendocrine stress

To aid interpretation of the complex metabolic dataset, exploratory correlation analyses were used to contextualize associations between bile acids, lipid species, and markers of renal-neurohormonal regulation (RAAS/vasopressin). Exploratory correlation analyses revealed preferential associations between bile acids, renin, and selected phosphatidylcholines in patients with PLE. Taken together, these patterns are consistent with an integrative hepato-renal-metabolic axis, reflecting coordinated metabolic stress across hepatic, renal, and lipid regulatory pathways rather than a defined mechanistic relationship. Alterations in bile acid profiles were among the most prominent features differentiating FPLE from non-PLE Fontan patients. Reduced levels of conjugated bile acids and their associations with renin and phosphatidylcholines suggest disruption of bile acid synthesis or trafficking in the setting of protein loss. Bile acids are increasingly recognized as signalling molecules influencing vascular tone, inflammation, and water homeostasis^51^, providing a potential mechanistic link between hepatic metabolism and renal–neurohormonal activation (RAAS/vasopressin)^52^.

Marked elevation of copeptin, a stable surrogate of vasopressin, supports activation of central neuroendocrine stress pathways in FPLE^53^. Together with increased renin and aldosterone levels, this pattern underscores pronounced renal–neurohormonal regulation and systemic homeostatic stress as defining features of the PLE phenotype. Vasopressin-mediated effects on fluid balance and vascular tone are consistent with key clinical characteristics of PLE in Fontan physiology, including volume imbalance and circulatory vulnerability. In this context, elevated copeptin is best interpreted as an integrative indicator of renal–neurohormonal activation rather than a disease-specific or standalone biomarker.

### Limitations and future directions

This study is limited by its observational design, modest cohort size, and the relatively small number of patients with PLE. Given these constraints, most analyses were necessarily unadjusted and exploratory in nature, and the observed associations may therefore be influenced by confounding related to clinical characteristics, disease severity, and treatment-related factors. In particular, detailed information on dietary intake and disease-specific nutritional interventions - which may influence lipid metabolism and bile acid profiles - was not systematically available and could therefore not be accounted for. Metabolomic, multivariate, and ROC analyses were also exploratory, and no internal validation procedures were applied; therefore, confirmation in independent cohorts will be essential to establish robustness and clinical utility.

From a systems-level perspective, the findings generate hypotheses regarding coordinated dysfunction across hepatic, lymphatic, renal, and renal–neurohormonal regulatory systems (RAAS/vasopressin) in Fontan-associated PLE. Alterations in bile acid profiles, potentially influenced by gut microbiome composition and intestinal barrier integrity, may represent one component of this integrated metabolic stress response. Future prospective studies combining longitudinal metabolomics with inflammatory markers, hemodynamic assessments, targeted endocrine profiling, and advanced hepatic and lymphatic imaging will be required to clarify temporal relationships, validate candidate biomarkers, and assess prognostic relevance.

Future studies should include prespecified marker selection and independent validation to assess generalizability and clinical relevance of candidate biomarker ratios identified in this exploratory analysis.

## Conclusion

Pediatric Fontan patients with PLE exhibit a distinct metabolic phenotype characterized by systemic protein loss, dyslipidemia, renal-neurohormonal activation (RAAS/vasopressin), altered amino acid handling, and bile acid disturbances. These findings indicate that metabolic alterations in FPLE extend beyond intestinal protein loss and reflect coordinated dysfunction across hepatic and endocrine regulatory systems. Composite metabolite ratios represent promising candidate markers for the identification and monitoring of PLE and warrant validation in larger, independent Fontan cohorts.

## Supplementary Information

Below is the link to the electronic supplementary material.

Supplementary Material 1

Supplementary Material 2

Supplementary Material 3

Supplementary Material 4

Supplementary Material 5

## Data Availability

The original contributions presented in this study are included in the article and its supplementary material. Further information is available from the corresponding author upon reasonable request.
