# Supplementary figures and images for: Fontan associated protein-losing enteropathy is linked to distinct metabolic and hepatic alterations
Source: Sci Rep. 2026 Feb 5;16:5256. doi: 10.1038/s41598-026-37974-1 (PMC12881532; doi:10.1038/s41598-026-37974-1)

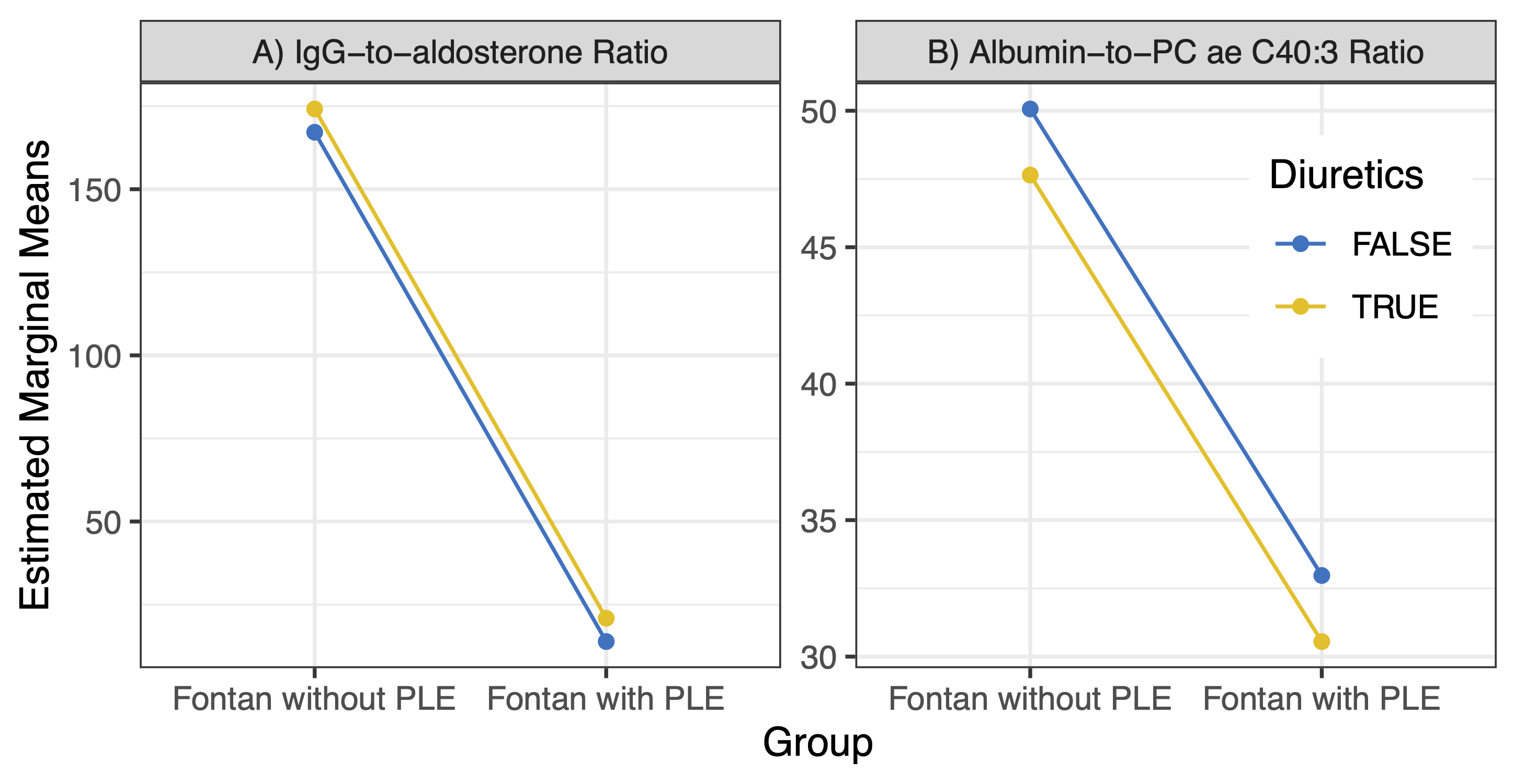

Supplement: Supplementary file 1 — Supplementary Material 1 [file 41598_2026_37974_MOESM1_ESM.png]

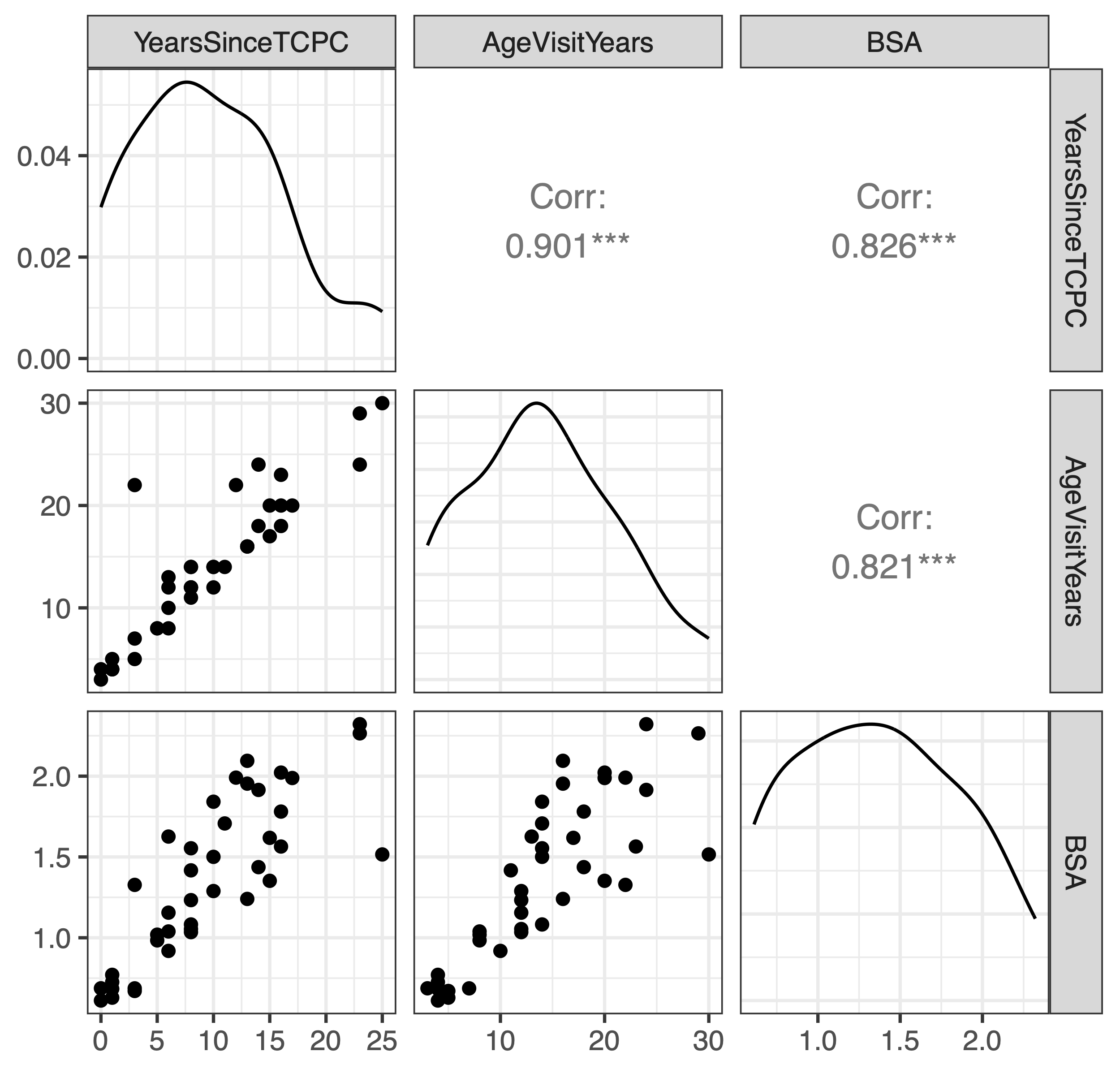

Supplement: Supplementary file 2 — Supplementary Material 2 [file 41598_2026_37974_MOESM2_ESM.png]
