# Supplementary material for: Fontan associated protein-losing enteropathy is linked to distinct metabolic and hepatic alterations
Source: Sci Rep. 2026 Feb 5;16:5256. doi: 10.1038/s41598-026-37974-1 (PMC12881532; doi:10.1038/s41598-026-37974-1)
Supplement: Supplementary file 3 — Supplementary Material 3 [file 41598_2026_37974_MOESM3_ESM.docx]

| **Term** | **IgGtoAldoRatio** | | **AlbtoPCaeC403Ratio** | |
| --- | --- | --- | --- | --- |
|  | **Estimate (95% CI)** | **pval** | **Estimate (95% CI)** | **pval** |
| AgeVisitYears | 1.53 (-8.21-11.27) | 0.758 | -0.58 (-1.55-0.38) | 0.238 |
| YearsSinceTCPC | 0.04 (-10.32-10.40) | 0.994 | 0.31 (-0.72-1.34) | 0.555 |
| Sex:Male | -18.38 (-81.47-44.70) | 0.568 | 2.46 (-3.81-8.72) | 0.442 |
| BSA | 25.91 (-103.29-155.11) | 0.694 | 4.45 (-8.39-17.30) | 0.497 |
| Diuretics:TRUE | -4.82 (-103.68-94.04) | 0.924 | -3.47 (-13.34-6.41) | 0.491 |
|  | Conditional R^2^ = 0.610  Marginal R^2^ = 0.033 | | Conditional R^2^ = 0.672  Marginal R^2^= 0.046 | |

**Supplementary Table S1.** Results of the comprehensive linear mixed-effects models including age at visit, years since TCPC, sex, body surface area, and diuretic therapy. Conditional and marginal R² values are reported. Effect estimates are shown to demonstrate consistency with the simplified primary models. Legend: AgeVisitYears indicates age at study visit in years. YearsSinceTCPC denotes the time elapsed since completion of the total cavopulmonary connection. Sex is coded as male versus female. BSA represents body surface area calculated at the time of examination. Diuretics indicates current diuretic therapy at the time of sampling (yes/no).
