# Supplementary material for: Fontan associated protein-losing enteropathy is linked to distinct metabolic and hepatic alterations
Source: Sci Rep. 2026 Feb 5;16:5256. doi: 10.1038/s41598-026-37974-1 (PMC12881532; doi:10.1038/s41598-026-37974-1)
Supplement: Supplementary file 4 — Supplementary Material 4 [file 41598_2026_37974_MOESM4_ESM.docx]

**Supplementary Table S3**. Exploratory correlation analysis between phosphatidylcholine species and bile acids in Fontan patients with and without protein-losing enteropathy. Pearson correlation coefficients (r) and corresponding p-values are displayed. Color is applied selectively for visual guidance: blue highlights correlation coefficients with |r| ≥ 0.6, and red highlights p-values < 0.05. Correlation analyses were exploratory and not adjusted for multiple testing.
