# Supplementary material for: Fontan associated protein-losing enteropathy is linked to distinct metabolic and hepatic alterations
Source: Sci Rep. 2026 Feb 5;16:5256. doi: 10.1038/s41598-026-37974-1 (PMC12881532; doi:10.1038/s41598-026-37974-1)
Supplement: Supplementary file 5 — Supplementary Material 5 [file 41598_2026_37974_MOESM5_ESM.docx]

**Supplementary Table S2.** Exploratory correlation analysis between renin, angiotensin II, bile acids, and selected clinical parameters in Fontan patients with and without protein-losing enteropathy. Pearson correlation coefficients (r) and corresponding p-values are shown. Color is applied selectively for visual guidance: blue highlights correlation coefficients with |r| ≥ 0.6, and red highlights p-values < 0.05. Correlation strength may be interpreted as very weak (r ≈ 0.2) to very strong (r ≥ 0.8). Analyzed parameters include conjugated and unconjugated bile acids, systolic blood pressure, and serum sodium and potassium. Correlations are presented for descriptive purposes and were not adjusted for multiple testing.
